# Common-onset masking simulated with a distributed-code model

**DOI:** 10.2478/v10053-008-0012-5

**Published:** 2008-07-15

**Authors:** Bruce Bridgeman

**Affiliations:** Department of Psychology, University of California, Santa Cruz, Ca. USA

**Keywords:** masking, metacontrast, lateral inhibition, mathematical model, object substitution, common-onset masking, backward masking, attention

## Abstract

A distributed-coding model incorporating lateral inhibition in a simulated nerve network has been successful in accounting for many properties of backward masking (Bridgeman, 1971, 1978), linking modeling with neurophysiology and psychophysics. Metacontrast is a variety of backward masking that is of particular interest in uncovering properties of visual coding because target and mask do not overlap in time or space, and it is the first stimulus that is reduced in visibility, not the second. The lateral inhibitory model can also simulate common-onset masking, where a target and mask appear simultaneously but the mask disappears after a variable delay, and it can reproduce qualitatively the effects of attention on object substitution by varying the time interval over which sensory codes are analyzed.

## INTRODUCTION

How is sensory information coded and processed in the brain? Our understanding of the answer to this question will be in terms of theories of brain function, theories that can be instantiated in mathematical models. Successful models will simulate real behavior and experience, and they will consist of parts that are identifiable with known brain structures. It is here that the development of useful models can begin.

Neuroanatomy can be described as a series of layers of neurons linked by parallel connections (Bridgeman, 1989, Ch. 2). Within these layers, neurons inhibit one another, a definition of lateral inhibition (Ratliff, 1965) that is known to take place at several levels in the afferent visual system. It is distinct from forward inhibition, where neurons inhibit neurons in a subsequent layer, and backward inhibition, where a more peripheral layer is inhibited.

The implications of lateral inhibition for sensory coding are not yet completely worked out, however. The inhibition does more than just suppress activity – it also normalizes output, so that the output of a layer undergoing lateral inhibition is less affected by the gross level of afferent activity than the input to that layer (Bridgeman, 1971). This point was later elaborated by Grossberg (1973) . Lateral inhibition also restructures the coding of afferent sensory information, as will be explored below.

### Application to metacontrast

In metacontrast (Stigler, 1910), a target is adjacent to a non-overlapping mask that is often of equal energy. If target and mask are presented briefly and simultaneously, both are seen. But if the mask’s appearance is delayed by about 50-100 ms, the target is no longer visible. It is a form of backward masking, so named because the effect seems to operate backward in time. Because the target and mask do not overlap either in time or in space at the peak of masking, the phenomenon promises to provide insight into both spatial and temporal aspects of visual coding. This masking is also described as ‘B-type’ masking, or U-shaped masking (referring to the shape of the mask-precedes-target part of the masking function).

A simple ‘busy signal’ model of the sort often invoked for forward masking can be eliminated immediately as an explanation for metacontrast, because it is the first stimulus that is masked, not the second. In these models, an incoming stimulus occupies processing resources so that a second stimulus that arrives before the processing of the first one is complete does not get processed (Arnell & Jolicoeur, 1999).

The first models of metacontrast invoked a few neurons; one slowly conducting neuron sensed the target, while a faster-conducting neuron sensed the mask (Weisstein, 1968). At a subsequent neural layer, the fast ‘mask’ signal caught up to the slow ‘target’ signal and inhibited it by forward inhibition. Simulations showed that a simple, mathematically analyzable nerve network could simulate backward masking (reviewed by Breitmeyer, 1984). Breitmeyer and Ganz (1976) later suggested a similar 2-stage architecture, again relying on differing conduction speeds in different channels and with a single cell as the hypothesized output, but without a mathematical model.

A model’s linking hypothesis is the output of the model that eventually links to perception. For Weisstein, the output of a single ‘detector’ neuron or feature detector coded the presence of a perceived object. The idea seemed to fit well with the feature detectors described in the visual systems of the cat and monkey. Problems with coding by feature detectors soon appeared, however (Weisstein, 1972). How could the brain identify novel objects with existing detectors, and who looks at the activities of the detectors to decide what is present?

### Distributed coding

An alternative to the feature detector scheme is distributed coding (Pribram, 1971), where it is not the gross level of activity of one or a group of neurons that codes a meaningful visual stimulus, but rather the combinations of activities of a large number of neurons. The combinatorics of this scheme are so much more efficient than the detector idea that its advantages become compelling even for relatively small neural nets. Consider the simplified case of binary,

on-off detectors. Detecting 1024 distinct states with these detectors, for example, requires 1024 neurons, and a subsequent layer that must know the meaning of each of the 1024 messages. A distributed code, however, can handle the same message with just 10 neurons assembled as a 10-bit binary number. Efficiency increases 100-fold. As the number of detectable objects increases, the economies of distributed coding become even more extreme.

Modeling of distributed codes followed quickly on the theory. A lateral inhibitory model of visual masking (Bridgeman, 1971) started with simulation of very general consequences of lateral inhibition for information coding in neural networks. Stimulating a neuron in a layer of simulated neurons linked by lateral inhibition causes a reduction in the activity of the neuron’s neighbors. But the neighbors of those cells, experiencing less inhibition, will increase their activity. The next set of neighbors will be more inhibited and will decrease their activity, and so on. Because the inhibition requires a delay, the result is a series of damped oscillations that proceed from the original point of disturbance like ripples in a pond. Eventually the whole pond’s activity is changed by the single disturbance.

One can no longer talk of feature detectors in this environment, because now stimulus-specific information is distributed across the relative activities of a large number of neurons. More complex stimuli will yield more complex patterns of excitation and inhibition, because each edge or contour in the image elicits an extensive series of waves. Each wave pattern is specific to the stimulus that elicited it; neuron-by-neuron illustrations of network states demonstrating this are given in Bridgeman (1971) . In the resulting coding, any stimulus entering the network eventually becomes coded (with varying information density) over the entire network.

A new linking hypothesis accompanies the new coding. If a stimulus changes activity across an entire network, then the presence of the stimulus must be coded in the network-wide pattern rather than in a particular cell. The identity of an incoming stimulus can be found by comparing the new activity with the activity elicited by other known stimuli. In the model used here this is done with squared correlations, reflecting the proportion of variance in the nerve net’s activity that is attributable to a particular stimulus. High correlations indicate the presence of the target stimulus, while low correlations signal masking.

This coding scheme is different from feature detectors because no particular neuron’s activity is identified with a particular stimulus – it is the pattern that is important. Correlation is a way to measure the similarity of two patterns of stimulation, in the case of masking a target-alone pattern and a target-mask pattern, to identify whether and when activity attributable to a target stimulus remains present in the modeled nerve net.

These ideas are incorporated in a computer simulation of a lateral inhibitory nerve net. The scheme has been successful in modeling a number of properties of metacontrast masking (Bridgeman 1971, 1978, 2001). It was also the most successful of a group of mathe-matical models in simulating a variation on backward masking, where target and mask were temporally contiguous and the mask was varied in duration (Di Lollo, von Mühlenen, Enns & Bridgeman, 2004).

### Simultaneous-onset and object substitution masking

In the 1960s and 1970s it was thought that stimulus onset asynchrony (SOA) was the critical timing variable in backward masking. Subsequent work, however, has identified interstimulus interval (ISI) and stimulus termination asynchrony (STA) as more important (Francis, Rothmayer & Hermens, 2004). A new masking paradigm, simultaneous-onset, brought a new challenge for mathematical modelers (Di Lollo, Bischof & Dixon, 1993). This paradigm presents a target and mask with geometries similar to metacontrast designs. They appear simultaneously, and the mask disappears after the target with a varying delay. Bischof and Di Lollo (1995) showed that metacontrast masking could be obtained with a simultaneous-onset paradigm.

If target and mask onset and offset are simultaneous, the target remains visible (identical to the zero-SOA condition of conventional metacontrast designs), but masking strengthens as the mask offset is delayed after the target offset. The target remains masked indefinitely as the mask offset is delayed further. The masking is weak if only one target and mask are presented, but becomes stronger as attention must be divided among larger numbers of masks in an array, with only one accompanied by a target.

Di Lollo, Enns and Rensink (2000) have extended this masking to object substitution, and have maintained that feed-forward or one-layer models cannot account for such a result, but Francis & Hermens (2002) used Weisstein’s original 1968 model, the Bridgeman (1978) model, and a model by Francis (1997) to simulate functions similar to those obtained psychophysically by Di Lollo et al. (2000).

Di Lollo, Enns and Rensink (2002) criticized the simulations, because Francis & Hermens had simulated stronger attention by weakening the mask energy. In the strongest attention condition there was no mask energy at all, and unsurprisingly there was also no masking. The simulations did show, however, that some of the properties of object substitution masking could be simulated with existing mathematical models and without reentrant processing, challenging the conclusion of Di Lollo et al. (2002) that object substitution includes “an early process affected by physical factors such as adapting luminance and a later process affected by attentional factors”. The questions addressed here are whether the attentional factors can be modeled independently of mask intensity, and whether the resulting masking tracks the psychophysical results.

## NEW SIMULATIONS

### Method

**Figure 1.**
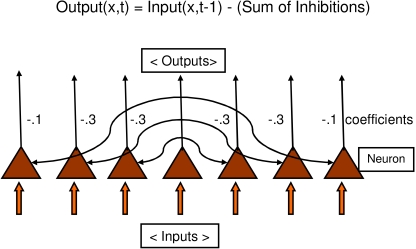
Design of the lateral inhibitory nerve net. Coefficients K1 to K3 define the fraction of a neuron’s output that is relayed to inhibit neighboring neurons. Stimulus presence is modeled as the activity over the entire 30-neuron net, of which connections of 1 neuron and a sample of 7 neurons are shown here.

The lateral inhibitory model is based on a linear array of 30 neurons, each with an input from a stimulus layer, an output to a response layer, and inhibition of its nearby neighbors (figure 1). Each neuron sends inhibition to 6 of its immediate neighbors, 3 on each side. The immediate neighbors receive inhibition with a strength *K*1 equal to 0.3 of the neuron’s output. The next pair of neighbors receives inhibition with *K*2 = 0.3, and the final pair receives *K*3 = 0.1. A small amount of Gaussian noise is added to each neuron at each iteration, simulating neural noise.

The target was always composed of 4 equally stimulated neurons in the center of the array; the mask was 2 groups of 2 neurons flanking the target with a separation of 1 neuron. Each iteration of inhibitory interactions occupies 30 msec of simulated time.

These are the model parameters and stimulus sizes used to simulate metacontrast masking with the model (Bridgeman, 1978; 2001). Durations of target and mask in the current simulations are 1 iteration of inhibition, representing 30msec of real time, except where noted below. The program is that of Francis (2003) , with changes as noted below to simulate novel conditions.

### Constant-intensity condition

**Figure 2.**
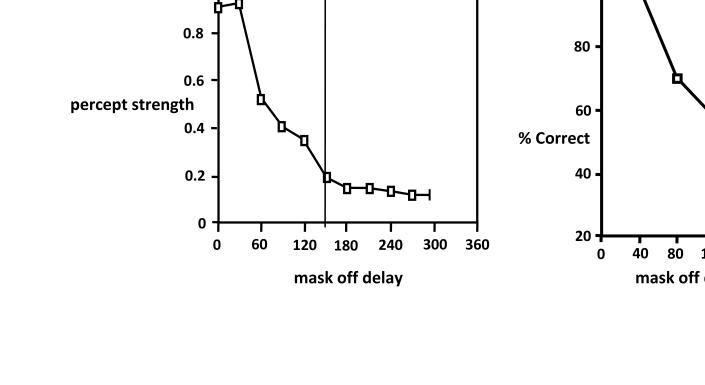
Object-substitution masking with the lateral inhibitory model, uncompensated for intensity. Left: Simulation, in 30msec increments, extended to 300msec after target offset. Right: Psychophysical results in 40msec increments to 160msec after target offset, replotted from data of Di Lollo et al. (1993). The vertical line in the simulation graph marks the time of the end of the psychophysical data.

Object-substitution masking was simulated with a constant mask intensity for each masking curve, so that increasing the duration of the mask also increases its total energy. Figure 2 (left) shows the result. Masking is somewhat stronger than in the strongest masking condition of Francis and Hermens (2002) because their strongest mask was only 0.25 times as strong as the target, whereas in figure 2 the target and mask are of equal intensity. On the right side of the figure are the psychophysical data of Di Lollo et al. (1993).

The simulation shows a brief period without masking, as do the psychophysical data, followed by a rapid decrease in visibility. The correlational response measure can never reach 1, since noise is added at each iteration. Thus higher correlations indicate greater percept strength, and lower correlations lower strength, in an environment where perfect correlation is impossible.

Because the mask’s intensity remained constant, its energy became stronger and stronger as the delay of mask offset increased. Thus it is not surprising that masking becomes stronger with increasing delay – the mask became stronger and stronger, while the target’s energy remained constant.

### Compensated-intensity condition

 What happens when the modeled mask intensity is compensated, its intensity becoming lower as its duration becomes longer? This compensation procedure was used by Di Lollo et al. (2000) ; apparent mask brightness was held constant while duration was increased, taking advantage of the intensity-duration reciprocity of Bloch’s law. Any increases in masking with mask duration could not be explained by energy considerations. Di Lollo et al. (2004) were also successful in using this technique to model masking with temporally contiguous target and mask, as reviewed above. 

The critical problem in modeling object-substitution masking is to simulate changes in the degree of attention. The psychophysical work manipulated attention by changing the number of simultaneously presented masks, only one of which contained a target, forcing subjects to distribute their attention over many masks. Francis & Hermens (2002) manipulated attention by adjusting mask intensity without changing target intensity, a procedure that Di Lollo et al. (2002) criticize because mask intensity in the psychophysical work was not changed as attention was manipulated. But the lateral inhibitory model already contains a parameter that can be used to simulate attention.

The reasoning begins with the fact that responses to attended stimuli are normally faster than responses to unattended stimuli of the same physical strength. The lateral inhibitory model requires that nerve net activity be integrated over several iterations, introducing a time delay in the neural code that represents a stimulus. Because an attended stimulus requires a faster response, it would be integrated over fewer iterations than a less well-attended stimulus that is responded to with a greater latency. Thus the number of iterations over which nerve-net activity is collected can serve to simulate the degree of attention given to a stimulus. At the same time, the model allows mask intensity to be compensated as mask duration increases.

Object substitution masking was simulated for three intervals of integration, 4, 8, and 12 iterations. At each duration, the intensity of the mask was adjusted by an amount derived from the psychophysical compensation factors used by Di Lollo et al. (2000) . 

**Figure 3.**
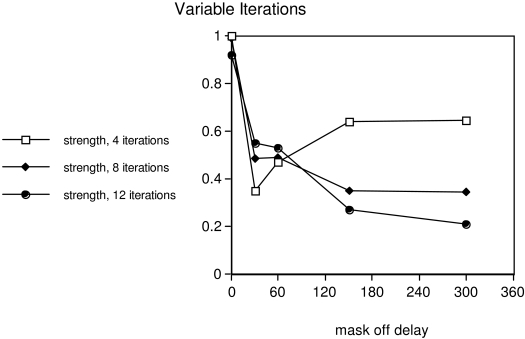
With identical stimulus parameters, simulations are run for 4, 8, or 12 iterations of lateral inhibition. In each case, mask intensity is adjusted as its duration is varied to match psychophysically derived equal-brightness stimulation. Total mask duration is 30msec longer than indicated on the horizontal axis, because target and mask appear simultaneously.

Results of the simulation are shown in figure 3. Except for an single point at 30 msec on the 4-iteration curve, simulating high attention, where activity is lower than the corresponding psychophysical function, the results correspond to those of Di Lollo et al. (2000) , experiment 1. The simulation of the high-attention condition (open squares in figure 3) has a dip in visibility followed by a partial recovery, just as the psychophysical results showed.

Since Di Lollo et al. began their delayed mask at 40msec delay, the deeper dip found here at 30msec might have occurred in the psychophysical data as well, if sampled at the shorter mask duration. As available attentional resources decrease, simulated by longer integration time with no change in the stimuli, the masking becomes stronger and the partial recovery disappears.

The simulation reproduces the most important properties of object substitution masking. In contrast to the brief period of no masking seen in figure 2, the masking functions begin their decline immediately both in this simulation and in the psychophysical data.

### Simultaneous offset

One problem in this simulation project is that perhaps the brightness compensation procedure is not enough, and a mask of long enough duration will always elicit strong masking, regardless of other considerations. As they work their way through the model nerve net, the damped oscillations elicited by the mask might eventually dominate the net’s activity at any reasonable stimulus amplitude.

This problem also concerned Di Lollo et al. (2000), but it could be resolved. According to those authors, “it cannot be said that masking occurs because the brief target is overwhelmed by the longer mask (e.g., the longer stimulus might be weighted more heavily or be given greater prominence in perceptual processing). This option is denied by the fact that no matter how long the mask or how brief the target, masking never occurs if the display begins with the mask alone and ends with a simultaneous display of target and mask”.

**Figure 4.**
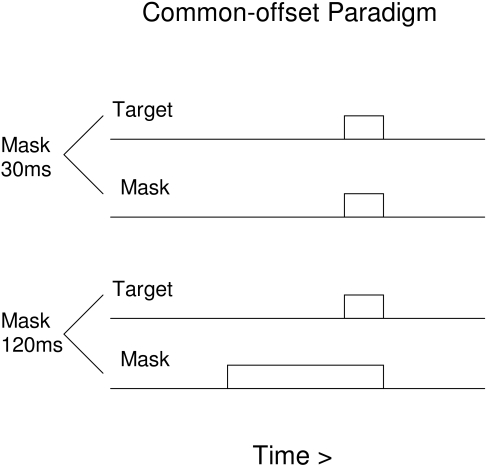
Paradigm for common-offset masking, showing the longest and shortest masks simulated.

This psychophysical finding can also test the lateral inhibitory model. To simulate simultaneous-offset masking, the parameters of the Francis (2003) instantiation of the lateral inhibitory model were modified to allow the mask to begin before the target (figure 4). A target was always presented for one iteration. The mask terminated along with the target, but it began either at the same time or at 30, 60 or 90msec before the target.

Masking is constant regardless of an increase in mask duration by a factor of four, without brightness compensation – mask intensity is the same at all durations. Modeled percept strength varies over the narrow range from 0.55 to 0.52 as the mask duration grows fourfold. Thus, in agreement with psychophysical observations, a strengthening of masking is not inevitable as the mask begins to dominate the total energy in the stimulus array. However, there is some masking; the model predicts that a careful psychophysical study to back up the informal observation of Di Lollo et al. (2000) would find some degree of masking at all mask durations.

## DISCUSSION

The prediction of Di Lollo et al. (2000) that an explanation of object substitution masking will require re-entrant processes appears to have been contradicted, as the single-layer lateral inhibitory model can account for most of the psychophysically measured masking effects. The model can be interpreted in at least two ways, however, with different implications for instantiation in the brain.

**Figure 5.**
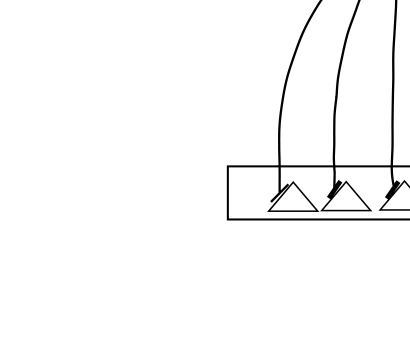
A two-layer interpretation of the architecture of the lateral inhibitory model.

The interpretation of this model until now has been as a single layer, with lateral inhibitory interactions between neighboring neurons within that layer. Another interpretation notes that the model’s neurons can be linked by inhibitory interneurons that could just as well be physically located in a subsequent processing layer, so that their inhibitory actions would be anatomically re-entrant on the model’s input neurons (figure 5). This sort of re-entrant processing is very simple, however, involving a single synapse and a direct return of activity to the original processing layer. It does not require complex interactions with other information at more central levels, normally thought of as top-down influences on perception.

Now that the behavior of lateral inhibition has been investigate in a number of situations, it is appro- priate to revisit the mechanisms by which masking takes place. At the first iteration of a target stimulus with the nerve net all of the net’s activity is driven by bottom-up connections, so that no masking can take place unless the target and mask overlap in space and time or a strong mask precedes the target. Lateral inhibition has most of its subsequent effect at the edges, because the normalization noted at the start of this paper suppresses responses to areas of uniform stimulation. After a few iterations, most of the target-specific activity is coded in regions just beyond the target’s edges; a mask presented in this region at this time interferes with that activity, and masking results. If the mask is introduced later, when the target’s representation has spread to many neurons, interference with the small area of the mask has less effect. This is the standard metacontrast condition.

In object substitution (figure 3), with the briefest integration condition the interactions are similar to those in standard metacontrast; when target and mask offset are close together in time, the mask interferes with the target’s spreading activity, but with larger mask delay the target is already firmly coded in redundant activity of many neurons when the mask appears. Four iterations of activity are not enough to allow the mask to dominate. With longer integration intervals, however, damped oscillations emanating from target offset and mask offset mix together in the network, interfering with one another and preventing target-like activity from reasserting itself. Since the mask remains present, it continues to exert a strong effect on total network activity. These qualitative descriptions are no substitute for mathematical modeling, of course, but hopefully they give a flavor of the sorts of interactions that lateral inhibition creates.

## Acknowledgements

This research was supported by a faculty research grant to Bruce Bridgeman from the academic senate of the University of California, Santa Cruz.
